# A transcriptomic analysis of cerebral microvessels reveals the involvement of Notch1 signaling in endothelial mitochondrial-dysfunction-dependent BBB disruption

**DOI:** 10.1186/s12987-022-00363-7

**Published:** 2022-08-26

**Authors:** Min Joung Lee, Jiebo Zhu, Jong Hun An, Seong Eun Lee, Tae Yeon Kim, Eungseok Oh, Yea Eun Kang, Woosuk Chung, Jun Young Heo

**Affiliations:** 1 Department of Biochemistry, Chungnam National University School of Medicine, Daejeon, 35015 Republic of Korea; 2 Brain Korea 21 FOUR Project for Medical Science, Chungnam National University, Daejeon, 35015 Republic of Korea; 3 Infection Control Convergence Research Center, Chungnam National University School of Medicine, Daejeon, 35015 Republic of Korea; 4 Research Center for Endocrine and Metabolic Disease, College of Medicine, Chungnam National University, Daejeon, 35015 Republic of Korea; 5 Division of Endocrinology and Metabolism, Department of Internal Medicine, Chungnam National University School of Medicine, Deajeon, 35015 Republic of Korea; 6 Bio-Synergy Research Center, Daejeon, 34141 Republic of Korea; 7 Department of Neurology, Chungnam National University Hospital, Daejeon, 35015 Republic of Korea; 8 Department of Anesthesiology and Pain Medicine, Chungnam National University School of Medicine, Daejeon, 35015 Republic of Korea; 9 Department of Anesthesiology and Pain Medicine, Chungnam National University Hospital, Daejeon, 35015 Republic of Korea

**Keywords:** Blood–brain barrier, Endothelial cell, Mitochondria, Intracellular stroke

## Abstract

**Background:**

Endothelial cells (ECs) in cerebral vessels are considered the primary targets in acute hemorrhagic brain injuries. EC dysfunction can aggravate neuronal injuries by causing secondary inflammatory responses and blood–brain barrier (BBB) disruption. Previous studies have reported that enhancement of mitochondrial function within ECs may reduce BBB disruption and decrease the severity of acute brain injuries. However, the molecular signaling pathways through which enhanced EC mitochondrial function is enhanced to exert this BBB protective effect have not been fully elucidated.

**Methods:**

To identify signaling pathways involved in linking EC-specific mitochondrial dysfunction and BBB disruption, we first performed RNA sequencing using isolated cerebral vessels from TEKCRIF1 KO mice, a mouse strain that displays EC-specific mitochondrial dysfunction. After identification, we assessed the significance of candidate signaling pathways using an intracerebral hemorrhage (ICH) mouse model. BBB integrity was assessed using an IgG leakage assay, and symptomatic changes were evaluated using behavioral assays.

**Results:**

Transcriptome analyses of the TEKCRIF1 KO mouse revealed significant changes in Notch1 signaling, a pathway intimately involved in BBB maintenance. We also observed a decrease in Notch1 signaling and expression of the mitochondrial oxidative phosphorylation (OxPhos) complex in the ICH mouse model, which also exhibits BBB disruption. To further assess the function of Notch1 signaling in relation to BBB disruption, we injected ICH model mice with adropin, a protein that interacts with the Notch1 ligand NB-3 and activates Notch1 signaling. We found that adropin prevented BBB disruption and reduced the extent (area) of the injury compared with that in vehicle controls, in association with alteration of mitochondrial function.

**Conclusion:**

These results suggest that the Notch1 signaling pathway acts as an upstream regulator of DEGs and can be a target to regulate the changes involved with endothelial mitochondrial dysfunction-dependent BBB disruption. Thus, treatment methods that activate Notch1 may be beneficial in acute brain injuries by protecting BBB integrity.

**Supplementary Information:**

The online version contains supplementary material available at 10.1186/s12987-022-00363-7.

## Background

Brain endothelial cells (ECs), a component of the neurovascular unit, which also includes neurons, astrocytes, smooth muscle cells and pericytes, constitute the BBB [1, 2], which functions in the active transport of essential nutrients to the brain while blocking brain access to harmful substances in peripheral blood [3]. Transcriptomic analysis of brain ECs has been suggested as a strategy for investigating possible pathways underlying BBB dysfunction in different neurological disease models [4–6]. Angiogenic processes in ECs prevent secondary damage arising from cerebrovascular impairments, such as ischemic disorder and hemorrhage (ICH), after acute primary brain injury [7, 8]. Endothelial phosphatase and tensin homolog (PTEN)/AKT signaling regulates BBB permeability by suppressing transcytosis [9]. However, the signaling pathway that modulates the accompanying mitochondrial defect in ECs and approaches for enhancing endothelial function to protect the BBB still need investigation.

ECs comprising the BBB are known to have a higher mitochondrial volume compared with peripheral ECs [2]. ECs require a tremendous amount of energy—supplied by mitochondrial respiration—to accomplish their innate function as a BBB component [10]. Mitochondrial function induced by inhibition of the mitochondrial respiratory chain complex aggravates the consequences of BBB disruption in an ischemic stroke model, increasing infarct volume [11]. As we recently reported, TEKCRIF1 KO mice, with EC-specific deletion of the mitochondrial OxPhos-related gene, *Crif1*, also known as *Gadd45gip1* (encoding GADD45G-interacting protein 1), display profound BBB defects accompanied by reduced expression of junctional proteins in ECs [12]. Although studies strongly suggest that mitochondrial energy metabolism in ECs is critical for maintaining BBB function, regulatory signaling pathways involved in BBB maintenance in the context of EC dysfunction have not been fully elucidated.

One representative neurological disease associated with BBB disruption is ICH, a cerebrovascular pathology that primarily occurs after stroke [13]. ICH is characterized by cerebral vascular deterioration involving BBB disruption, increased intracranial pressure through hematoma production, and hemolysis-induced neurotoxicity [14, 15]. Because mitochondrial dysfunction in ECs leads to secondary brain damage [11], it may also be a therapeutic target for reducing secondary brain impairment in ICH [16]. To overcome ICH-induced neuronal damage and achieve functional recovery, researchers have investigated treatment with the Nrf2 activator, dimethyl fumarate [17], and upregulation of the micro RNA, miR-137-3p, with the green tea extract, EGCG [18], which reduce or inhibit oxidative stress. Notwithstanding such suggested treatments for cerebrovascular disease, a mitochondrial-targeting modulator has not yet been reported. Thus, we investigated the modulatory signaling pathway underlying mitochondrial defects in ECs comprising the BBB.

In the present study, we used RNA-sequencing to evaluate endothelial-related molecular signaling in isolated brain microvessels from TEKCRIF1 KO mice. Ingenuity Pathway Analysis (IPA) revealed that Notch1 signaling is a potential upstream regulator of DEGs in *Crif1-*deficient brain microvessels. We specifically tested the hypothesis that Notch1 expression is decreased in ICH model mice in association with diminished mitochondrial OxPhos function and BBB disruption. We further demonstrated that activation of Notch1 signaling by treatment with adropin exerted a preventive effect against the development of ICH.

## Methods

### Animals

For RNA sequencing, we used TEK-cre, *Crif1*^+/+^ for control, as mentioned WT and TEK-cre, *Crif1*^flox/flox^, as mentioned TEKCRIF1 KO mouse which we previously published [12].

For ICH mouse mode, Male 8-week-old C57BL/6 mice were purchased from Dooyeol Biotech (South Korea). Mice were maintained in a controlled facility at 22–24 ℃ with a 12 h light–dark cycle. Animal experiments were approved by the Institutional Animal Care and Use Committee of Chungnam National University (Ethical approval number, 202103A-CNU-022).

### Isolation of brain microvessels

The mouse brain tissue was immediately transferred to MCDB 131 medium on ice (Thermo Fisher, 10372019). After homogenizing the brain tissue, centrifuge at 2000*g* for 5 min at 4 °C. The pellet was resuspended in 15% dextran/PBS (Signa-Aldrich, 31390), and then the samples were centrifuged at 10,000*g* for 15 min at 4 °C. After the pellets were washed using 1 ml of PBS, transferred to a 40-μm cell strainer with 10 ml PBS. The microvessels were collected using 0.5% BSA/MCDB 131 medium and then centrifuged at 5000 *g* for 10 min at 4 °C [19].

### RNA preparation, library preparation, and sequencing

RNA extraction from isolated brain microvessels was performed (three samples per WT and KO group) using TRIzol (Thermo Tisher Scientific) following the manufacturer’s protocol. RNA sequencing was performed by Ebiogen (Seoul, Republic of Korea). RNA samples were checked RNA purity (260/280 > 1.8) and RNA integrity number (RIN) value by Agilent 2100 Bioanalyzer (Agilent Technologies, Amstelveen, The Netherlands) and used RNA samples with RNA values that are greater than or equal to 7.0. RNA quantification was performed using an ND-2000 spectrophotometer (Thermo Inc., DE, USA). The NEBNext Ultra II Directional RNA-Seq Kit (NEW ENGLAND BioLabs, Inc., UK) was used to prepare libraries from total RNA. Quantification was performed using the library quantification kit using a StepOne Real-Time PCR System (Life Technologies, Inc., USA). High-throughput sequencing was performed as paired-end 100 sequencing using HiSeq X10 (Illumina, Inc., USA).

### Bioinformatics transcriptome analysis

Quality control of raw reads resulting from sequencing was performed using FastQC. Adapter and low-quality reads (< Q20) were removed using FASTX_Trimmer and BBMap. The trimmed reads were mapped to the reference genome using TopHat. The levels of gene expression were estimated using FPKM (Fragments per kilobase of exon per million reads) values and RPKM (Reads per kilobase of transcript per million mapped reads). For differentially expressed gene (DEG) analysis, we performed the “DESeq2” R package. Gene set enrichment analysis (GSEA) was performed using the “fgsea” R package. Gene sets included the KEGG pathway (Kyoto Encyclopaedia of Genes and Genomes) and C5 GOBP-Notch signaling pathway gene sets from MSigDB (v7.5.1, http://software.broadinstitute.org/gsea) using obtained DEG with p-value < 0.05 and |fold change|≥ 2. For GSEA analysis used KEGG pathway analysis, the results represented in the graph were significantly up-or down-regulated in the KO group based on normalized enrichment score (NES). Heat maps were generated by the R program. Ingenuity Pathway Analysis (IPA) (Qiagen) was used for canonical pathways and upstream regulators related to genes. The RNA sequencing raw data are available at the National Center for Biotechnology Information (NCBI) Gene Expression Omnibus (GEO) database with accession number: GSE207106.

### Protein extraction and Western blot

Proteins from isolated brain microvessels and brain tissue were extracted using RIPA buffer with protease inhibitor and phosphatase inhibitor cocktail (Roche Applied Science, Vienna, Austria) described previously [12]. Protein samples were separated by SDS-PAGE and transferred to the PVDF membrane (Millipore, Burlington, MA, USA). Membranes were blocked with 5% skim milk for 1 h and then incubated at 4 ℃, overnight with specific primary antibodies against Crif1 (1:1000, sc-134882, Santa Cruz Biotechnology, CA, USA), Notch1 (1:1000, 3608, Cell Signaling, MA, USA), Hes1 (1:500, sc-166410. Santa Cruz Biotechnology, CA, USA), Total OXPHOS complex (1:1000, ab110413, Abcam), adropin (1:500, NBP1-26387, Novus biologicals, CO, USA), β-actin (1:1000, sc-47778, Santa Cruz Biotechnology, CA, USA). After washing three times for 10 min with TBS/T, membranes were incubated with secondary antibodies (1:1000, Sigma-Aldrich, St. Louis, MO, USA) for 2 h at room temperature. Membranes were washed, and signals were observed using an ECL solution (WEST-ZOL). Images were quantified with ImageJ software.

### Surgical procedures for the ICH model and treatments

Stereotaxic Z-axis was modified at 40° from the vertical state to avoid motor cortex damage by needle insertion [20]. 8-week-old C57BL/6 mice were anesthetized using sevoflurane and put on a stereotaxic frame (DAVID KOPF INSTRUMENTS, CA, USA). After a small incision in the skin, collagenase type VII-S (0.5U, Sigma, St. Louis, MO) was injected (0.2 µl/min, AP + 0.4 mm, ML + 3.8 mm from bregma and DV -3.2 mm from the skull) using 33 G blunt needle. The needle was left for 5 min and removed, then sutured the skin. The mice were allowed to recover. Adropin (Novopro, Shanghai, China) was dissolved in saline and adropin (900 nmol/kg) was administered by intraperitoneal injection at 1 h after collagenase injection.

### Measurement of hematoma volume

Brain tissues of mice injected collagenase were isolated and fixed with 4% paraformaldehyde. Brains were sliced into five 1-mm-thick coronal sections. Sections were scanned and then analyzed hematoma volume by ImageJ.

### Immunohistochemistry staining

Eight-week-old C57BL/6 mice and ICH model mice were perfused and fixed with 4% paraformaldehyde for IgG staining. The brains were dehydrated in 30% sucrose solution and then frozen and sliced using a cryotome. The sections were washed with PBS for 5 min and incubated with 2.5% blocking serum (VECTOR lab, CA, USA) for 20 min. after that, the sections were incubated with Goat-anti-mouse IgG antibody (H + L), and biotinylated (VECTOR lab, CA, USA) for 30 min. After washing for 5 min with PBS, incubated in peroxidase substrate solution (Dako North America Inc., USA) until the desired stain intensity develops, about 10 s. The sections were washed and then mounted on a slide glass. For cresyl violet staining, the brain tissue sections were put on a slice glass and incubated with 0.2% cresyl violet for 5 min at 37 ℃. After washing with water and 90% EtOH, the sections were dehydrated and mounted.

### Immunofluorescence staining

Isolated microvessels from the brain were resuspended in PBS and put 50 µl on a micro slide glass and air-dry and then permeabilized the microvessels with 0.1% NP-40 in PBS for 15 min. After blocking the samples with 5% BSA in PBS for 1 h, the samples were incubated overnight at 4 ℃ with primary antibodies; anti-CD31(Millipore, CA, USA), anti-NDUFA9 (Abcam, CA, USA), anti-Crif1 (Santa Cruz Biotech, USA). After washing with 0.1% NP-40 in PBS three times, incubated the samples with secondary antibodies conjugated with Alexa Fluor 488, 594, and 647 (Jackson, PA, USA) for 1 h at room temperature. After washing, the samples were air-dried and then mounted using a fluorescent mounting solution (Dako North America Inc., USA). The samples were imaged using Leica confocal microscope (Leica, Bensheim, Germany).

### Open-field test

A Mouse was placed in a 40 × 40 × 40-cm box respectively. The movement was recorded for 10 min. Velocity and total moved distance were analyzed by EthoVision XT 11.5 software.

### Cylinder test

Each mouse was placed in a cylinder (diameter 12 cm, height 20 cm). The movement was recorded for 10 min. Forelimb-use asymmetry was assessed by quantifying the percentage of impaired and non-impaired forelimb use; contralateral paw use = (right-left)/(right + left) × 100 [21].

### Analysis of oxygen consumption rate with isolated mitochondria

The striatum region (AP + 2.4 mm ~ AP + 0.8 from bregma) was prepared for isolation of mitochondria. After separating the brain tissue next to the hematoma region, the tissue was homogenized in mitochondrial isolation buffer (70 mm sucrose, 210 mM mannitol, 5 mM, HEPES, 1 mM EGTA, 0.5% fatty acid-free BSA, pH 7.2) on ice. The samples were centrifuged (600 *g*, 5 min, 4 °C), and the supernatants were transferred to new tube and re-centrifuged (17,000 *g*, 10 min, 4 °C). The pellets were suspended with mitochondrial isolation buffer and the concentration of isolated mitochondria were analyzed. Next, isolated mitochondria were diluted in mitochondrial assay buffer (70 mM sucrose, 220 mM mannitol, 10 mM KH2PO4, 5 mM MgCl2, 2 mM HEPES, 1 mM EGTA, 0.2% fatty acid-free BSA, 10 mM succinate, 2 mM rotenone, pH 7.2) and seeded in an XF-24 plate (25 ug per well, Agilent, CA, USA). OCR analysis was performed at 37 °C using the XF24 analyzer (Agilent, CA, USA). Basal respiration was measured twice before measuring OCR after sequential injection of ADP, oligomycin, CCCP and antimycin A.

### Statistics

All results are presented as mean ± standard deviation (SD). The statistical significance of all the experimental data was analyzed by a two-tailed unpaired Student’s *t-test* and one-way ANOVA followed by Games-Howell’s multiple comparison test using GraphPad Prism 8 (GraphPad Software Inc., San Diego, CA, USA). The statistical significance of all the data is indicated by *P < 0.05, **P < 0.01, ***P < 0.001 (Vehicle vs. ICH), #P < 0.05 (ICH vs. ICH + Adropin).

## Result

### Isolation and identification of cerebral microvessels in the TEKCRIF1 KO mouse

**Fig. 1:**
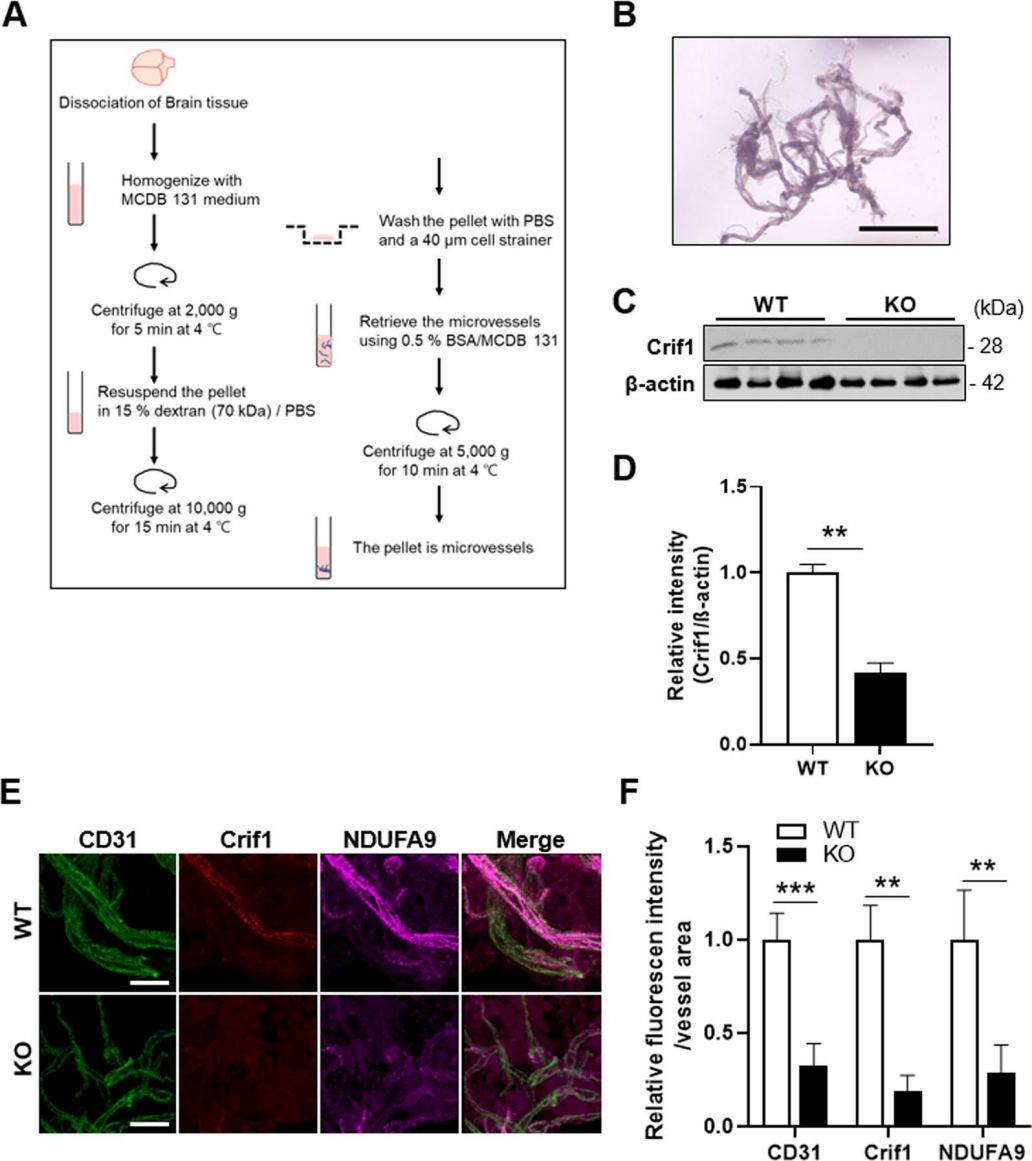
Mitochondrial dysfunction in isolated brain microvessels from TEKCRIF1 KO mice. **A** Schematic depiction of methods for isolating microvessels from the brain. **B** Morphology of isolated brain microvessels from a TEKCRIF1 WT mouse. Scale bar: 100 μm. **C**, **D** Western blot analysis showing the decrease in Crif1 protein in KO mice (n = 7 mice/group). **E**, **F** Immunofluorescence staining showing the decrease in Crif1, NDUFA9 (mitochondrial complex I subunit), and CD31 (EC marker) in isolated microvessels from TEKCRIF1 KO mice (n = 5 mice/group 6 slides per group). Scale bar: 20 μm. Immunofluorescence staining intensity was quantified using ImageJ. Data are presented as means ± SD from three independent experiments performed under the same conditions (**P < 0.01, ***P < 0.001 vs. WT)

To identify molecular changes in ECs that accompany mitochondrial defects, we first isolated microvessels from brain tissue of TEKCRIF1 mice following the process shown schematically in Fig. 1A and described in Methods [19]. The morphology of the intact microvessels is shown in Fig. 1B. To verify *Crif1* deletion in microvessels, we performed Western blotting of isolated microvessels using an anti-Crif1 antibody. As expected, Crif1 protein expression was decreased by more than 60% in TEKCRIF1 KO mice compared with WT mice (Fig. 1C, D). Consistent with Western blotting results, immunofluorescence analyses revealed a decrease in the intensity of Crif1 fluorescence in microvessels in TEKCRIF1 KO mice. These analyses further showed a decrease in colocalization of NDUFA9 (mitochondrial complex I subunit) with the EC marker CD31 in TEKCRIF1 KO mice compared with WT mice (Fig. 1E, F). Thus, ECs in microvessels isolated from TEKCRIF1 KO mice showed a decrease in a mitochondrial marker upon Crif1 deletion.

### Transcriptomic analyses of isolated brain microvessels from TEKCRIF1 KO mice reveal a decrease in BBB-maintenance-related genes

**Fig. 2:**
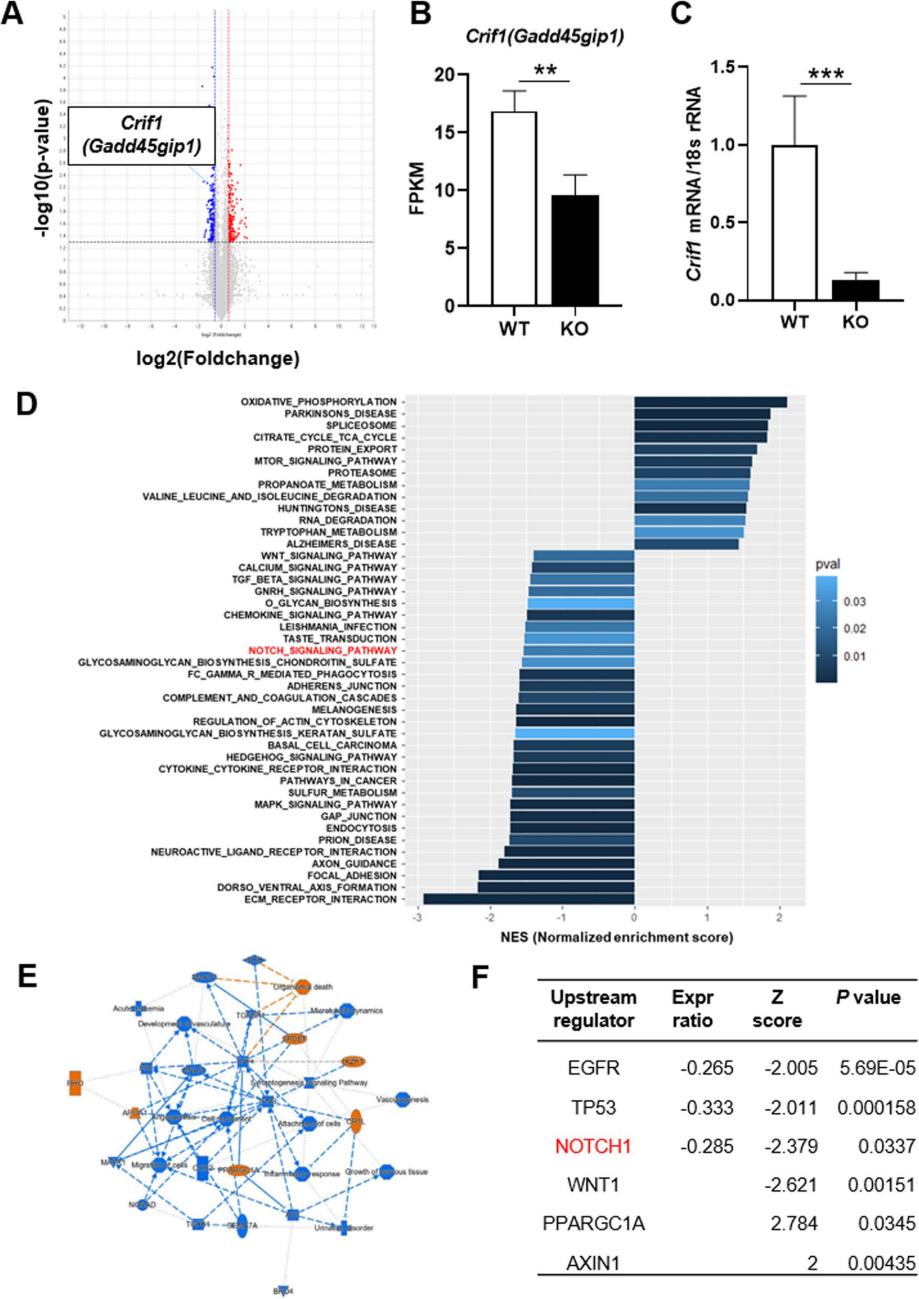
Transcriptomic analysis of isolated brain microvessels from TEKCRIF1 KO mice. **A** Volcano plot showing differentially expressed genes in isolated cerebral microvessels from TEKCRIF1 KO mice compared with those from WT mice (n = 3 mice/group). **B** FPKM values for the *Crif1* (*Gadd45gip1*) gene. **C** *Crif1* mRNA level, analyzed by qPCR (n = 6 mice/group). Data are presented as means ± SD (**P < 0.01, ***P < 0.001 vs. WT). (D) Up- and down-regulated pathways in isolated brain microvessels from TEKCRIF1 KO mice. Pathways were categorized using the KEGG pathway database. **E** Network analysis by IPA. Blue denotes inhibited pathways and orange denotes activated pathways. **F** List of most potent upstream regulators related to *Crif1* deletion in ECs identified through IPA upstream regulator analysis

To identify relevant regulatory signaling pathways underlying the mitochondrial defect caused by the *Crif1* gene deficiency in ECs, we performed RNA sequencing on microvessels isolated from brain tissue of TEKCRIF1 mice. First, to validate our microvessel preparation, we confirmed the cellular composition by comparing FPKM (fragments per kilobase of exon per million reads mapped) values for the EC marker genes *Pecam1* (platelet/endothelial cell adhesion molecule 1) and *Tjp1* (tight junction protein 1), astrocyte marker *Aqp4* (aquaporin 4), and pericyte marker *Anpep* (alanyl aminopeptidase, membrane) (Additional file 1: Fig. S1A). This analysis showed that isolated microvessels are composed of ECs with astrocytes and pericytes, and that their composition was not significantly different between WT and KO mice. A volcano plot analysis showed a decrease in *Crif1 (Gadd45gip1)* expression in microvessels from TEKCRIF1 KO mice compared with those from WT mice, consistent with FPKM values and *Crif1* mRNA levels (Fig. 2A–C, Additional file 1: Fig. S1B). RNA sequencing identified a total of 1,119 differentially expressed genes (*p*-value < 0.05) (Additional file 1: Fig. S1C). We then used gene set enrichment analysis (GSEA) in conjunction with the KEGG pathway database to investigate correlated signaling pathways (Fig. 2D). Down-regulated signaling pathways identified included extracellular matrix (ECM) receptor and actin cytoskeleton regulation, which correlate with maintenance of BBB structure [22–24]. Other down-regulated pathways identified included cell survival signaling pathways, such as MAPK [25] and Wnt pathways, which are involved in promoting angiogenesis [25, 26]. Interestingly, in addition to the expected pathways, we found that Notch1 signaling, which is known as a pathway for blood vessel homeostasis [27], was down-regulated. A network analysis (IPA), performed to further identify the regulatory mechanism and key factors involved in mitochondrial-dysfunction-related BBB disruption in ECs (Fig. 2E) showed that vasculature development and angiogenesis were down-regulated in TEKCRIF1 KO mice, as we observed in our previous paper [12]. Moreover, this analysis further established that down-regulation of Notch1, a powerful upstream transcriptional regulator, exerts a significant (*p*-value < 0.05) inhibitory influence on correlated pathway-related molecules with high absolute-value Z scores, including EGFR (epidermal growth factor receptor), TP53 (tumor protein 53), PPARGC1A (peroxisome proliferative activated receptor, gamma, coactivator 1 alpha), and WNT1 (Wnt family member 1). These results are consistent with experimental RNA sequencing results showing the Expr ratio (Fig. 2F, Additional file 2: Table 1). Taken together, these findings indicate that the Notch1 signaling pathway is a candidate mechanism for regulating BBB maintenance in the context of EC mitochondrial dysfunction.

### Expression levels of Notch1-signaling-related genes and protein are decreased in TEKCRIF1 KO mice

**Fig. 3:**
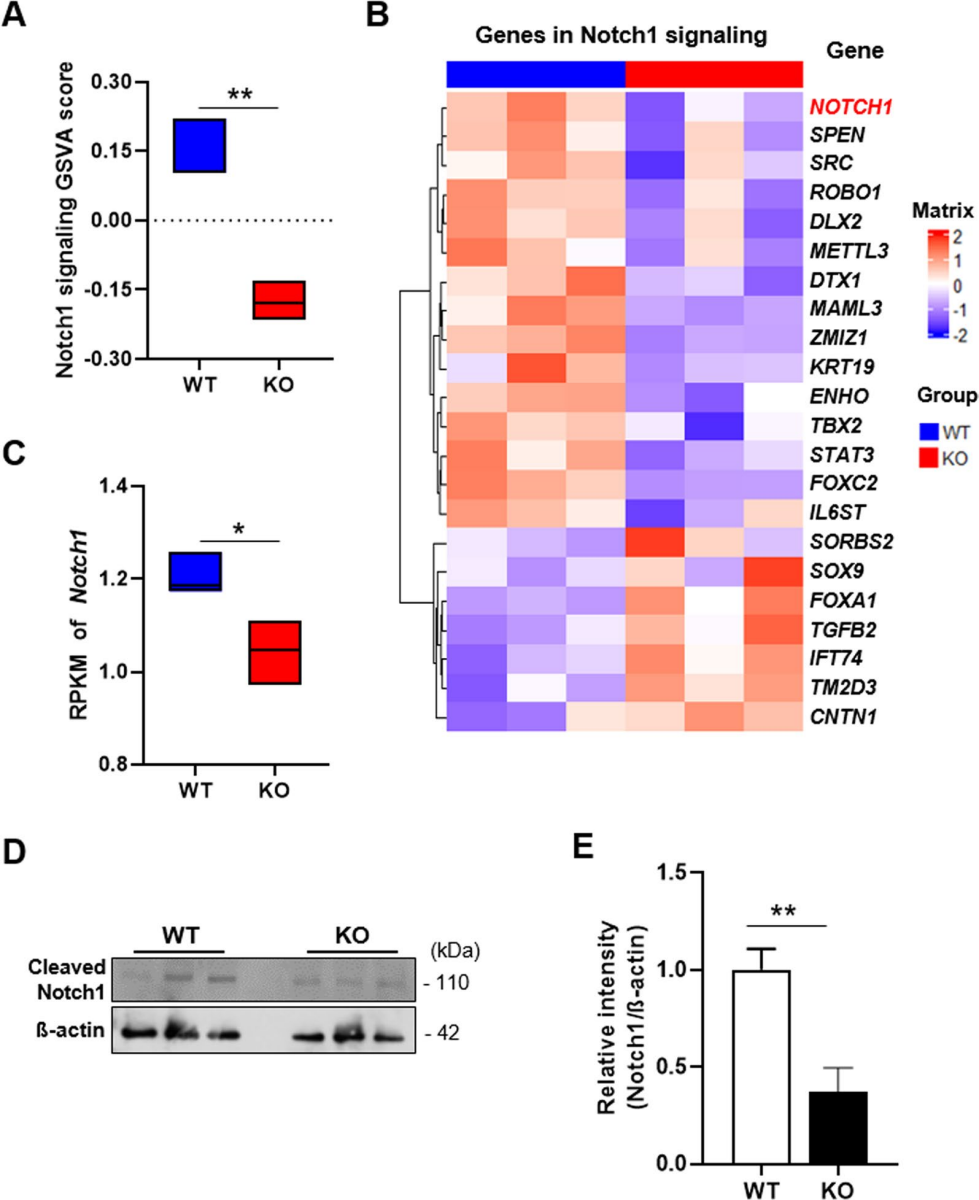
Gene set analysis of Notch1 signaling as a key regulator of BBB maintenance. **A** Comparison of GSVA scores of Notch1 signaling pathway genes. **B** Heatmap of Notch1 signaling pathway gene expression levels, determined by analyzing fold change (|FC|≥ 1.2) and p-value (≤ 0.1), in WT and KO groups (**C**) RPKM value of the Notch1 gene. Data are presented as means ± SD (*P < 0.05, **P < 0.01, ***P < 0.001 vs. WT). **D**, **E** Notch1 protein level in isolated brain microvessels from the TEKCRIF1 mouse (n = 8 mice). Data are presented as means ± SD from three independent experiments performed under the same conditions (*P < 0.05, **P < 0.01, ***P < 0.001 vs. WT)

We next performed an in-depth analysis of the involvement of Notch1-related genes using a gene set variation analysis (GSVA), which indicates the degree of coordinated expression of genes in this pathway. A total of 166 Notch1 signaling pathway-related genes showed a tendency toward decreased expression in TEKCRIF1 KO mice compared with WT mice (Additional file 1: Fig. S1D). The GSVA score obtained using differentially expressed Notch signaling-related genes (|fold change|≥ 1.2, *p*-value ≤ 0.05) was significantly decreased in isolated brain microvessels from TEKCRIF1 KO mice comparing with those from WT mice (Fig. 3A). Next, we investigated expression of the Notch1 signaling pathway gene set by analyzing fold change (|FC|≥ 1.2) and *p*-values (< 0.1) in WT and KO groups, presented as a heatmap (Fig. 3B). The RPKM (reads per kilobase of transcript per million mapped reads) value of Notch1 was significantly decreased in microvessels from TEKCRIF1 KO mice compared with those from WT mice (Fig. 3C). To verify that changes in Notch1 protein expression were consistent with transcriptomic analyses, we performed Western blotting on isolated brain microvessels from TEKCRIF1 KO and WT mice. Notch1 protein level was decreased in microvessels from TEKCRIF1 KO mice compared with those from WT mice (Fig. 3D, E). Collectively, these observations suggest that Notch1 is a candidate upstream regulator of DEGs and may be a target to regulate the changes involved with endothelial mitochondrial dysfunction-dependent BBB disruption.

### Notch1 expression is decreased in the injured area in association with BBB disruption in an ICH mouse model

**Fig. 4:**
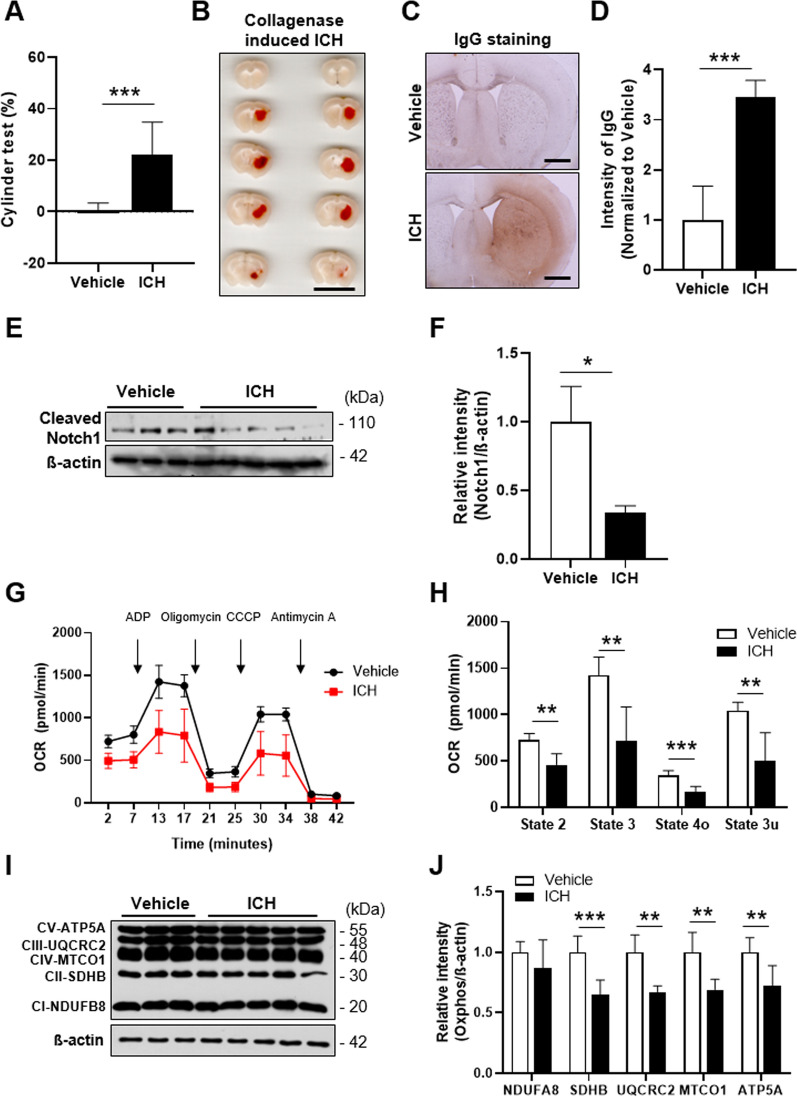
Pathophysiology of the ICH model and decreased Notch1 signaling in ICH model mice. **A** Cylinder test performed 24 h after collagenase injection (n = 10 mice/group). **B** Representative brain sections showing hematoma volume in the collagenase-induced ICH mouse model. Scale bar: 10 mm. **C** Representative coronal brain sections showing IgG staining, used to evaluate BBB disruption. Scale bar: 100 μm. **D** Intensity of IgG staining, quantified using ImageJ (n = 3 mice, 7 slides per group). Decreased Notch1 (**E**) and Hes1 (**F**) protein levels in ICH model mice (n = 6 for Vehicle, n = 10 mice for ICH group). **G** Mitochondrial respiration in the striatum of Vehicle and ICH groups by OCR analysis (n = 6 mice/group). **H** OCR analysis values. State 2: basal respiration; State 3: after ADP injection; State 4o: after oligomycin injection; State 3u: after CCCP injection. **I**, **J** Total OxPhos complex protein levels. Data are presented as means ± SD from three independent experiments performed under the same conditions (*P < 0.05, **P < 0.01, ***P < 0.001 vs. Vehicle)

ICH is a cerebrovascular pathology characterized by a large hemorrhage inside the brain parenchyma accompanied by BBB disruption [28, 29]. To identify the effect of the Notch1 signaling pathway in BBB protection, we generated an ICH mouse model. Since the striatal area affects motor behavior [30], we first evaluated general movement activity 24 h after collagenase injection using an open-field test. ICH injury resulted in about a 16% decrease in distance moved and movement velocity compared with vehicle-treated control mice (Additional file 1: Fig. S2A, B). We also assessed locomotor asymmetry produced by injury to the right hemisphere using a cylinder test [31] and found prominently decreased use of the left paw, indicating forelimb asymmetry in the ICH model (Fig. 4A). Following assessment of behavioral defects, we measured hematoma volume in the injured brain area 24 h after injury (Fig. 4B). Similar to previously reported results [20, 32], the average volume of hematoma in the ICH model was approximately 23 mm^3^ (Additional file 1: Fig. S2C). Secondary brain injury in the ICH model, produced as a result of hemorrhage-induced toxic effects, were evaluated by cresyl violet staining, which revealed a damaged area around the hematoma region in the striatum of ICH model mice (Additional file 1: Fig. S2D). To identify BBB disruption in the injured area, we performed immunostaining with IgG on cryo-sections of brain tissue [33]. As expected, the IgG-positive area increased around the region where collagenase was injected in the ICH model compared with the site of vehicle injection in the control group (Fig. 4C, D).

Based on transcriptomic analyses of the TEKCRIF1 KO mouse, we hypothesized that BBB disruption, which results in secondary brain injury in ICH, is correlated with changes in the Notch1 signaling pathway. To identify changes in this pathway, we isolated the striatal region around the hematoma lesion in the ICH mouse model and performed Western blotting. Consistent with transcriptomic analysis results, Notch1 protein expression was decreased by ~ 60% in the striatum around the hematoma lesion in the ICH group compared with the vehicle group (Fig. 4E, F). These results suggest that the Notch1 signaling pathway is down-regulated in brain areas of the ICH model where cerebrovascular function is impaired, and taken together with the results of transcriptomic analyses of the TEKCRIF1 KO mouse, support that idea that this pathway can be a therapeutic target for BBB maintenance.

### Mitochondrial dysfunction is a co-occurrence in the injured area of the ICH mouse brain

It is known that brain tissue in the ICH model is damaged by oxidative stress arising from iron-induced toxicity resulting from the lysis of red blood cells after BBB disruption [34, 35]. In addition, the rupture of blood vessels in the ICH model impairs oxygen delivery to brain cells, which can lead to mitochondrial dysfunction [16, 36]. Accordingly, we assessed mitochondrial function by measuring oxygen consumption rate (OCR) and analyzing mitochondrial OxPhos complex proteins in mitochondria isolated from the same region where changed Notch1 expression was observed. OCR, including basal respiration (state 2) and maximal respiration (state 3u), was lower in the ICH group than in the vehicle control group (Fig. 4G, H). Consistent with this, mitochondrial OxPhos complex II, III, IV, and V expression were decreased in the ICH group (Fig. 4I, J). These results demonstrate a mitochondrial defect in the region surrounding the hematoma and suggest that this contributes to the change in Notch1 expression.

### Reversal of ICH pathophysiology by injection of adropin

**Fig. 5:**
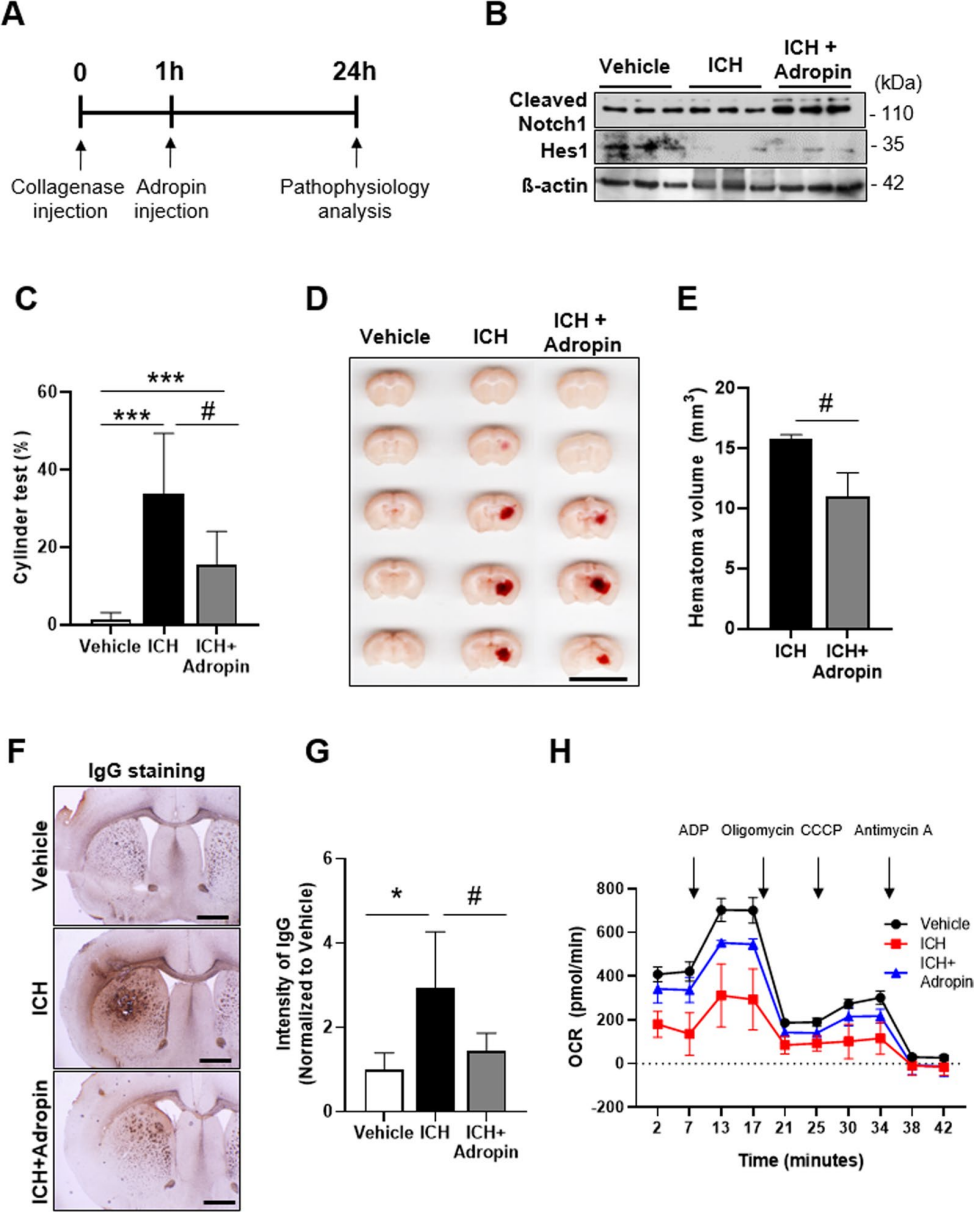
Adropin attenuates ICH pathology through activation of the Notch1 signaling pathway. **A** Experimental time-line for adropin injection in ICH model mice. **B** Attenuated Notch1 and Hes1 protein levels in the adropin-treated ICH group (n = 6 mice/group). **C** Cylinder test performed on ICH model mice after adropin injection (n = 11 mice/group). **D** Representative brain sections showing hematoma volume after adropin injection in an ICH model mouse. Scale bar: 10 mm. **E** Quantification of hematoma volume (n = 3 mice/group). **F** Representative coronal brain sections showing IgG staining after adropin injection. Scale bar: 100 µm. **G** IgG staining intensity, quantified using ImageJ (n = 5 mice, 8 slides per group). **H** Mitochondrial respiration in the striatum after adropin injection in ICH model mice, determined by OCR analysis (n = 4 mice/group). Data are presented as means ± SD from three independent experiments performed under the same conditions (*P < 0.05, **P < 0.01, ***P < 0.001, Vehicle vs. ICH; ^#^P < 0.05, ICH vs. ICH + adropin)

Interestingly, our TEKCRIF1 KO transcriptomic analysis showed that the RPKM value for the *Enho* (energy homeostasis associated) gene, which was included in Notch1 signaling pathway-related gene sets, was lower in TEKCRIF1 KO mice than in WT mice (Additional file 1: Fig. S3A). Notably, *Enho* encodes the protein adropin, which stimulates the Notch1 signaling pathway [37–39]. Thus, to determine whether activation of the Notch1 signaling pathway can rescue ICH-induced pathologies, including mitochondrial dysfunction, we treated ICH model mice with adropin. Adropin was administered 1 h after collagenase injection, based on a previous study that showed a gradual decrease in adropin expression after establishment of ICH and MCAO (middle cerebral artery occlusion) stroke mouse models (Fig. 5A) [38, 40]. First, we found that adropin expression was increased in adropin-treated ICH mice compared with that in the ICH-only group (Additional file 1: Fig. S3B, C). As expected, up-regulated expression of Hes1, which is a downstream protein in the Notch1 signaling pathway, increased the expression of cleaved Notch1 in the adropin-treated ICH group compared with the ICH-only group (Fig. 5B, Additional file 1: Fig. S5D). Compared with the ICH-only group, the adropin-treated group also showed increased motor behavior in the open-field test (Additional file 1: Fig. S3E), showing a 14% increase in moved distance and a 17% increase in movement velocity. Cylinder tests showed that adropin treatment enhanced motor coordination by ~ 45% compared with the ICH-only group (Fig. 5C). Consistent with these attenuated behavioral deficits, we found that hematoma volume was lower in the adropin-treated ICH group compared with that in the ICH-only group (Fig. 5D, E). IgG-positive areas corresponding to areas of BBB disruption were also decreased by adropin treatment (Fig. 5F, G).

Activation of the Notch1 pathway could enhance mitochondrial quality control by increasing the activity of the mitochondrial fusion/fission machinery [41, 42]. Moreover, our transcriptomics results suggest that the decrease in Notch1 signaling affects mitochondrial defects in the TEKCRIF1 KO mouse. To verify that the Notch1 signaling pathway can modulate mitochondrial respiration, we measured OCR and monitored expression of mitochondrial OxPhos complex components (Fig. 5H). Interestingly, adropin treatment partially recovered mitochondrial respiration, including basal respiration and state 3 (after oligomycin injection) compared with that observed in the ICH-only group (Additional file 1: Fig. S3F). Consistent with this, adropin treatment also attenuated OxPhos complex protein levels compared with those in the ICH-only group (Additional file 1: Fig. S3G). Taken together, these results indicate that adropin treatment partially rescues ICH-related behavioral deficits through enhancement of BBB integrity and mitochondrial respiration in ICH model mice via activation of Notch1 signaling.

## Discussion

Disruption and torsion of blood vessels in the brain have been regarded as initiators of acute brain injury, including ICH and traumatic brain injuries [43, 44]. Moreover, peripheral aggregated proteins known to be involved in neurodegenerative disease can accumulate and penetrate into the brain parenchyma through a dysfunctional BBB [45]. We previously reported that mitochondrial defects in ECs are closely related to the BBB disruption observed in the TEKCRIF1 KO mouse [12]. To further identify the regulatory signaling pathways involved in BBB maintenance in the context of EC dysfunction, we performed transcriptomic profiling of brain microvessels from TEKCRIF1 KO mice. We identified Notch1 as a candidate gene for BBB disruption, specifically as it relates to the accompanying mitochondrial defect in isolated microvessels from brain tissue of the TEKCRIF1 KO mouse. Furthermore, we discovered reduced activity of the Notch1 signaling pathway in a mouse model of the cerebrovascular pathology ICH, and that treatment with adropin, which stimulates the Notch1 signaling pathway, rescued ICH pathological phenotypes, including behavioral impairments and BBB disruption, while modestly alleviating mitochondrial respiration deficits.

ECs in blood vessel have different characteristics in different regions of the body [46]. In brain vessels, especially those that constitute the BBB, there is a much higher distribution of mitochondria compared with peripheral blood vessels [2]. Although it is well known that mitochondrial function deteriorates in stroke-induced animal models in which the cerebrovascular barrier is broken [11], why the volume of mitochondria in the brain is higher than that of peripheral blood vessel and how molecular mechanisms change to promote mitochondrial dysfunction in ECs have not been studied. A previous transcriptomics analysis on isolated brain ECs demonstrated that certain pathways are down-regulated in a stroke model, including cell adhesion, extracellular matrix organization, and Notch signaling [6]. Moreover, a transcriptomics analysis of purified ECs from the cerebral cortex during development showed down-regulation of a signaling pathway involved in mitochondrial dysfunction accompanied by changes in an angiogenic-related pathway [4]. In the current study, we focused on cerebral microvessels in which ECs showed profound mitochondrial dysfunction, and identified changes in Notch1, PPARGC1A and WNT1 by IPA analysis as mediators of BBB disruption and accompanying mitochondrial defect. On the basis of transcriptomic results, we go one step beyond reports that Notch1 signaling is involved in the angiogenesis process, suggesting that Notch1 signaling is the specific pathway most sensitive to changes in mitochondrial function in ECs constituting the BBB. Additionally, the role of WNT1 and PPARGC1A pathways will need to be further investigated in future studies.

Notch1 signaling is involved in heart development and repair through activation of EC development, differentiation, and proliferation [47]. Once Notch1 is activated by binding of its ligand, the Notch1 intracellular domain (NICD) is released and initiates the transcription of target genes, such as Hes1 [48]. Although it has been previously reported that changes in Notch1 activity are related to mitochondrial fusion and fission [41], the current study is the first to report that Notch1 signaling activity changes in isolated microvessels from transgenic mice (TEKCRIF1 KO) in the setting of EC mitochondrial dysfunction. In constructing an ICH model in which BBB disruption and mitochondrial dysfunction are considered characteristic pathologies, we also found an associated decrease in Notch1 signaling. Although the ICH pathologies originated from not only the endothelial level, mitochondrial damage in endothelial cells is a powerful pathogenic factor to initiate the secondary injury in ICH [16, 49, 50]. That is, a decrease in Notch1 signaling, which occurs in the early stages of ICH with BBB disruption, is associated with a deficiency in mitochondrial function and is therefore considered to be a factor that aggravates the pathology. Accordingly, we tested whether stimulating Notch1 signaling could prevent the pathology and accompanying mitochondrial deficiency. This possibility was confirmed by administration of adropin, which stimulates Notch1 signaling. Thus, a decrease in Notch1 signaling in a local area with a profound defect in mitochondrial function is a factor that aggravates ICH pathology with BBB disruption, and stimulating Notch1 signaling can maintain BBB integrity and attenuate mitochondrial dysfunction in the surrounding nervous tissue. Accordingly, adropin treatment can attenuate the deterioration associated with the pathology and could possibly prevent the development of ICH. Also, it can protect neuron cells which synthesize adropin [51] and the decrease of adropin in ICH could be alleviated since secondary brain injury decreased as we observed adropin expression in Additional file 1: Fig. S3. Even though our results are not consistent with a previous study, adropin has previously been shown to attenuate BBB disruption without effecting hematoma volume [38], this may be due to differences in the dose and injection site of collagenase. Also, the previous study used a much lower concentrations of adropin compared to the present study. As adropin is involved in to energy homeostasis and lipid metabolism and can regulate endothelial function [39, 52, 53], the effects of adropin in cerebral vessels must be further studied. Furthermore, adropin is currently suggested as a clinical biomarker of obesity and is reported to play a role in cardiovascular protection and type 2 diabetes mellitus [37, 54, 55]. Our findings are valuable in this context insofar as they provide support for clinical trials of adropin in patients with disruption of BBB and mitochondria, such as ICH disease. Therefore, the effect of adropin in cerebral vessels and cerebrovascular disease has to be studied in further future.

Focusing on mitochondrial dysfunction in ECs, we identified Notch1 signaling as a therapeutic target for BBB maintenance in TEKCRIF1 KO mice and demonstrated a beneficial effect of activating Notch1 signaling in an ICH model. However, the Notch1 signaling pathway is also known to be a molecular mechanism that coordinates cellular events by interacting and communicating with neighboring cells [56]. Because a transcriptomic study comparing single cultured ECs with ECs cocultured with pericytes—a cell type of the neurovascular unit—showed differences in BBB maintenance-related pathway that depended on the presence of neighboring cells [57], further studies are needed to investigate interactions between ECs and astrocytes and/or pericytes in the neurovascular unit. Taken together, our results suggest that Notch1 signaling is a key regulator under conditions in which mitochondrial function in ECs of the BBB is disrupted and strengthen the effect of Notch1 signaling as a therapeutic target for maintaining BBB integrity in cerebrovascular disease.

## Conclusions

In conclusion, we identified the Notch1 signaling pathway as a therapeutic target for BBB maintenance in TEKCRIF1 KO mice, which exhibit a mitochondrial defect in ECs. Activation of Notch1 signaling through treatment with adropin reduced ICH pathologies, including BBB leakage, in association with attenuation of mitochondrial function in brain tissue. Our findings suggest that Notch1 signaling is a key regulator in ECs of the BBB in the setting of mitochondrial dysfunction and strengthen the effect of Notch1 signaling as a therapeutic target for maintaining BBB integrity in cerebrovascular disease.

## Supplementary Information

**Additional file 1: Figure S1.** Differentially expressed genes in isolated brain microvessels from TEKCRIF1 KO mice, determined by RNA sequencing. **Figure S2.** Characteristics of the ICH mouse model. **Figure S3.** Rescue of ICH pathology by injection of adropin. **Figure S4.** Schematic summary of this study.

**Additional file 2: Table S1.** Information of Antibodies. **Table S2.** List of upstream regulators related to *Crif1* deletion in ECs, determined by IPA.

## Data Availability

Data analyzed using this study are included in this published article. The RNA sequencing data generated during this study are available at the National Center for Biotechnology Information (NCBI) Gene Expression Omnibus (GEO) database with accession number: GSE207106.

## References

[1] Tajes M, Ramos-Fernandez E, Weng-Jiang X, Bosch-Morato M, Guivernau B, Eraso-Pichot A (2014). The blood–brain barrier: structure, function and therapeutic approaches to cross it. Mol Membr Biol.

[2] Oldendorf WH, Cornford ME, Brown WJ. The large apparent work capability of the blood-brain barrier: a study of the mitochondrial content of capillary endothelial cells in brain and other tissues of the rat. Ann Neurol. 1977 May;1(5):409-17. 10.1002/ana.410010502 617259

[3] Kemper MF, Zhao Y, Duckles SP, Krause DN. Endogenous ovarian hormones affect mitochondrial efficiency in cerebral endothelium via distinct regulation of PGC-1 isoforms. J Cereb Blood Flow Metab. 2013 Jan;33(1):122-8. 10.1038/jcbfm.2012.159 PMC3597365 23093066

[4] Daneman R, Zhou L, Agalliu D, Cahoy JD, Kaushal A, Barres BA. The mouse blood-brain barrier transcriptome: a new resource for understanding the development and function of brain endothelial cells. PLoS One. 2010 Oct 29;5(10):e13741. 10.1371/journal.pone.0013741 PMC2966423 21060791

[5] Kalari KR, Thompson KJ, Nair AA, Tang X, Bockol MA, Jhawar N (2016). BBBomics-human blood brain barrier transcriptomics hub. Front Neurosci..

[6] Munji RA-O, Soung AA-O, Weiner GA-O, Sohet F, Semple BD, Trivedi A, et al. Profiling the mouse brain endothelial transcriptome in health and disease models reveals a core blood-brain barrier dysfunction module. (1546–1726 (Electronic)). 10.1038/s41593-019-0497-x PMC6858546 31611708

[7] Navaratna D, Guo S, Arai K, Lo EH (2009). Mechanisms and targets for angiogenic therapy after stroke. Cell Adh Migr.

[8] Zhu H, Zhang Y, Zhong Y, Ye Y, Hu X, Gu L (2021). Inflammation-mediated angiogenesis in ischemic stroke. Front Cell Neurosci..

[9] Cui Y, Wang Y, Song X, Ning H, Zhang Y, Teng Y, Wang J, Yang X. Brain endothelial PTEN/AKT/NEDD4-2/MFSD2A axis regulates blood-brain barrier permeability. Cell Rep. 2021 Jul 6;36(1):109327. 10.1016/j.celrep.2021.109327 34233198

[10] Zhou W, Zhang Y, Jiao Y, Yin W, Dong H, Xu S (2021). Dexmedetomidine maintains blood–brain barrier integrity by inhibiting Drp1-related endothelial mitochondrial dysfunction in ischemic stroke. Acta Biochim Biophys Sin.

[11] Doll DN, Hu H, Sun J, Lewis SE, Simpkins JW, Ren X (2015). Mitochondrial crisis in cerebrovascular endothelial cells opens the blood–brain barrier. Stroke.

[12] Lee MJ, Jang Y, Han J, Kim SJ, Ju X, Lee YL (2020). Endothelial-specific Crif1 deletion induces BBB maturation and disruption via the alteration of actin dynamics by impaired mitochondrial respiration. J Cereb Blood Flow Metab.

[13] Owens WB (2011). Blood pressure control in acute cerebrovascular disease. J Clin Hypertens.

[14] Leonardo CC, Robbins S, Dore S (2012). Translating basic science research to clinical application: models and strategies for intracerebral hemorrhage. Front Neurol.

[15] Jia P, He J, Li Z, Wang J, Jia L, Hao R (2021). Profiling of blood-brain barrier disruption in mouse intracerebral hemorrhage models: collagenase injection vs autologous arterial whole blood infusion. Front Cell Neurosci..

[16] Chen W, Guo C, Feng H, Chen Y. Mitochondria: Novel Mechanisms and Therapeutic Targets for Secondary Brain Injury After Intracerebral Hemorrhage. Front Aging Neurosci. 2021 Jan 27;12:615451. 10.3389/fnagi.2020.615451 PMC7873050 33584246

[17] Zhao X, Sun G, Zhang J, Ting S-M, Gonzales N, Aronowski J (2015). Dimethyl fumarate protects brain from damage produced by intracerebral hemorrhage by mechanism involving Nrf2. Stroke.

[18] Vilella R, Sgarbi G, Naponelli V, Savi M, Bocchi L, Liuzzi F (2020). Effects of standardized green tea extract and its main component, EGCG, on mitochondrial function and contractile performance of healthy rat cardiomyocytes. Nutrients.

[19] Lee YK, Uchida H, Smith H, Ito A, Sanchez T (2019). The isolation and molecular characterization of cerebral microvessels. Nat Protoc.

[20] Leclerc JL, Lampert AS, Diller MA, Immergluck JB, Dore S (2015). Prostaglandin E2 EP2 receptor deletion attenuates intracerebral hemorrhage-induced brain injury and improves functional recovery. ASN Neuro.

[21] Ruan J, Yao Y (2020). Behavioral tests in rodent models of stroke. Brain Hemorrhages.

[22] Hussain B, Fang C, Chang J. Blood-Brain Barrier Breakdown: An Emerging Biomarker of Cognitive Impairment in Normal Aging and Dementia. Front Neurosci. 2021 Aug 19;15:688090. 10.3389/fnins.2021.688090 PMC8418300 34489623

[23] Baeten KM, Akassoglou K. Extracellular matrix and matrix receptors in blood-brain barrier formation and stroke. Dev Neurobiol. 2011 Nov;71(11):1018-39. 10.1002/dneu.20954 PMC3482610 21780303

[24] Shi Y, Zhang L, Pu H, Mao L, Hu X, Jiang X (2016). Rapid endothelial cytoskeletal reorganization enables early blood-brain barrier disruption and long-term ischaemic reperfusion brain injury. Nat Commun.

[25] Bonni A, Brunet A, West AE, Datta SR, Takasu MA, Greenberg ME. Cell survival promoted by the Ras-MAPK signaling pathway by transcription-dependent and -independent mechanisms. Science. 1999 Nov 12;286(5443):1358-62. 10.1126/science.286.5443.1358 10558990

[26] Néstor T, Masckauchán H, Shawber CJ, Funahashi Y, Li C-M, Kitajewski J (2005). Wnt/β-catenin signaling induces proliferation, survival and interleukin-8 in human endothelial cells. Angiogenesis.

[27] Akil A, Gutiérrez-García AK, Guenter R, Rose JB, Beck AW, Chen H (2021). Notch signaling in vascular endothelial cells, angiogenesis, and tumor progression: an update and prospective. Front Cell Dev Biol..

[28] Wang J, Fields J, Doré S (2008). The development of an improved preclinical mouse model of intracerebral hemorrhage using double infusion of autologous whole blood. Brain Res.

[29] Yang J, Li Q, Wang Z, Qi C, Han X, Lan X (2017). Multimodality MRI assessment of grey and white matter injury and blood-brain barrier disruption after intracerebral haemorrhage in mice. Sci Rep.

[30] Nakamura T, Xi G, Hua Y, Schallert T, Hoff JT, Keep RF. Intracerebral hemorrhage in mice: model characterization and application for genetically modified mice. J Cereb Blood Flow Metab. 2004 May;24(5):487-94. 10.1097/00004647-200405000-00002 15129180

[31] Shi X, Bai H, Wang J, Wang J, Huang L, He M (2021). Behavioral assessment of sensory, motor, emotion, and cognition in rodent models of intracerebral hemorrhage. Front Neurol.

[32] Xu J, Chen Z, Yu F, Liu H, Ma C, Xie D (2020). IL-4/STAT6 signaling facilitates innate hematoma resolution and neurological recovery after hemorrhagic stroke in mice. Proc Natl Acad Sci.

[33] Natah S, Srinivasan S, Pittman Q, Zhao Z, Dunn J (2009). Effects of acute hypoxia and hyperthermia on the permeability of the blood-brain barrier in adult rats. J Appl Physiol (Bethesda, Md.:1985).

[34] Zhang Y, Khan S, Liu Y, Wu G, Yong VW, Xue M. Oxidative Stress Following Intracerebral Hemorrhage: From Molecular Mechanisms to Therapeutic Targets. Front Immunol. 2022 Mar 9;13:847246. 10.3389/fimmu.2022.847246 PMC8959663 35355999

[35] Hu X, Tao C, Gan Q, Zheng J, Li H, You C (2016). Oxidative stress in intracerebral hemorrhage: sources, mechanisms, and therapeutic targets. Oxid Med Cell Longev..

[36] Huang J, Jiang Q. Dexmedetomidine Protects Against Neurological Dysfunction in a Mouse Intracerebral Hemorrhage Model by Inhibiting Mitochondrial Dysfunction-Derived Oxidative Stress. J Stroke Cerebrovasc Dis. 2019 May;28(5):1281-1289. 10.1016/j.jstrokecerebrovasdis.2019.01.016 30797643

[37] Gao S, McMillan RP, Zhu Q, Lopaschuk GD, Hulver MW, Butler AA. Therapeutic effects of adropin on glucose tolerance and substrate utilization in diet-induced obese mice with insulin resistance. Mol Metab. 2015 Jan 17;4(4):310-24. 10.1016/j.molmet.2015.01.005 PMC4354928 25830094

[38] Yu L, Lu Z, Burchell S, Nowrangi D, Manaenko A, Li X (2017). Adropin preserves the blood-brain barrier through a Notch1/Hes1 pathway after intracerebral hemorrhage in mice. J Neurochem.

[39] Sato K, Yamashita T, Shirai R, Shibata K, Okano T, Yamaguchi M (2018). Adropin contributes to anti-atherosclerosis by suppressing monocyte-endothelial cell adhesion and smooth muscle cell proliferation. Int J Mol Sci.

[40] Yang C, Lavayen BP, Liu L, Sanz BD, DeMars KM, Larochelle J (2021). Neurovascular protection by adropin in experimental ischemic stroke through an endothelial nitric oxide synthase-dependent mechanism. Redox Biol.

[41] Dai S-H, Wu Q-C, Zhu R-R, Wan X-M, Zhou X-L (2020). Notch1 protects against myocardial ischaemia-reperfusion injury via regulating mitochondrial fusion and function. J Cell Mol Med.

[42] Zhou XL, Wu X, Xu QR, Zhu RR, Xu H, Li YY, Liu S, Huang H, Xu X, Wan L, Wu QC, Liu JC. Notch1 provides myocardial protection by improving mitochondrial quality control. J Cell Physiol. 2019 Jul;234(7):11835-11841. 10.1002/jcp.27892 30515819

[43] Al-Mufti F, Amuluru K, Changa A, Lander M, Patel N, Wajswol E, Al-Marsoummi S, Alzubaidi B, Singh IP, Nuoman R, Gandhi C. Traumatic brain injury and intracranial hemorrhage-induced cerebral vasospasm: a systematic review. Neurosurg Focus. 2017 Nov;43(5):E14. 10.3171/2017.8.FOCUS17431 29088959

[44] Lok J, Leung W, Murphy S, Butler W, Noviski N, Lo EH (2011). Intracranial hemorrhage: mechanisms of secondary brain injury. Acta Neurochir Suppl.

[45] Takata F, Nakagawa S, Matsumoto J, Dohgu S. Blood-Brain Barrier Dysfunction Amplifies the Development of Neuroinflammation: Understanding of Cellular Events in Brain Microvascular Endothelial Cells for Prevention and Treatment of BBB Dysfunction. Front Cell Neurosci. 2021 Sep 13;15:661838. 10.3389/fncel.2021.661838 PMC8475767 34588955

[46] Feng W, Chen L, Nguyen PK, Wu SM, Li G (2019). Single cell analysis of endothelial cells identified organ-specific molecular signatures and heart-specific cell populations and molecular features. Front Cardiovasc Med..

[47] Dabral S, Tian X, Kojonazarov B, Savai R, Ghofrani HA, Weissmann N (2016). Notch1 signalling regulates endothelial proliferation and apoptosis in pulmonary arterial hypertension. Eur Respir J.

[48] Takeshita K, Satoh M, Ii M, Silver M, Limbourg FP, Mukai Y (2007). Critical role of endothelial notch1 signaling in postnatal angiogenesis. Circ Res.

[49] Imai T, Iwata S, Hirayama T, Nagasawa H, Nakamura S, Shimazawa M (2019). Intracellular Fe2+ accumulation in endothelial cells and pericytes induces blood-brain barrier dysfunction in secondary brain injury after brain hemorrhage. Sci Rep.

[50] Wang Z, Zhou F, Dou Y, Tian X, Liu C, Li H (2018). Melatonin alleviates intracerebral hemorrhage-induced secondary brain injury in rats via suppressing apoptosis, inflammation, oxidative stress, DNA damage, and mitochondria injury. Transl Stroke Res.

[51] Shahjouei S, Ansari S, Pourmotabbed T, Zand R. Potential Roles of Adropin in Central Nervous System: Review of Current Literature. Front Mol Biosci. 2016 Jun 27;3:25. 10.3389/fmolb.2016.00025 PMC4921473 27446928

[52] Lovren F, Pan Y, Quan A, Singh KK, Shukla PC, Gupta M (2010). Adropin is a novel regulator of endothelial function. Circulation.

[53] Mushala BAS, Scott I (2021). Adropin: a hepatokine modulator of vascular function and cardiac fuel metabolism. Am J Physiol Heart Circ Physiol.

[54] TičinovićKurir T, Miličević T, Novak A, Vilović M, Božić J (2020). Adropin—potential link in cardiovascular protection for obese male type 2 diabetes mellitus patients treated with liraglutide. Acta Clin Croat.

[55] Canguven O, Talib RA, El Ansari W, Yassin DJ, Salman M, Al-Ansari A. Testosterone therapy has positive effects on anthropometric measures, metabolic syndrome components (obesity, lipid profile, Diabetes Mellitus control), blood indices, liver enzymes, and prostate health indicators in elderly hypogonadal men. Andrologia. 2017 Dec;49(10). 10.1111/and.12768 28295504

[56] Bigas A, Espinosa L (2016). Notch signaling in cell–cell communication pathways. Curr Stem Cell Rep.

[57] Heymans M, Figueiredo R, Dehouck L, Francisco D, Sano Y, Shimizu F, Kanda T, Bruggmann R, Engelhardt B, Winter P, Gosselet F, Culot M. Contribution of brain pericytes in blood-brain barrier formation and maintenance: a transcriptomic study of cocultured human endothelial cells derived from hematopoietic stem cells. Fluids Barriers CNS. 2020 Jul 28;17(1):48. 10.1186/s12987-020-00208-1 PMC7385894 32723387

