# Supplementary material for: A transcriptomic analysis of cerebral microvessels reveals the involvement of Notch1 signaling in endothelial mitochondrial-dysfunction-dependent BBB disruption
Source: Fluids Barriers CNS. 2022 Aug 26;19:64. doi: 10.1186/s12987-022-00363-7 (PMC9414148; doi:10.1186/s12987-022-00363-7)
Supplement: Supplementary file 1 — Additional file 1: Figure S1. Differentially expressed genes in isolated brain microvessels from TEKCRIF1 KO mice, determined by RNA sequencing. Figure S2. Characteristics of the ICH mouse model. Figure S3. Rescue of ICH pathology by injection of adropin. Figure S4. Schematic summary of this study. [file 12987_2022_363_MOESM1_ESM.docx]

**Additional information**

**Additional Figure S1**


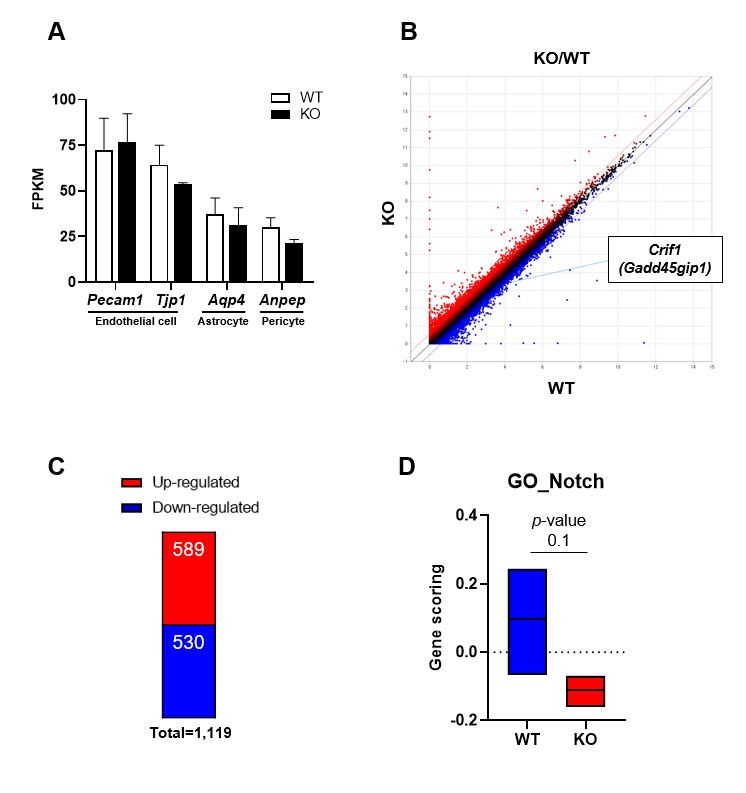


**Additional Figure S1. Differentially expressed genes in isolated brain microvessels from TEKCRIF1 KO mice, determined by RNA sequencing.**

(A) FPKM value of cellular markers (*Pecam1*, *Tjp1*: ECs; *Aqp4*: astrocytes; *Anpep*: pericytes) showing the cellular composition of isolated microvessels. (B) Scatter plot showing differentially expressed genes in isolated cerebral microvessels from TEKCRIF1 KO mice compared with those from WT mice (n = 3 mice/group). (C) Total number of significantly enriched KEGG gene sets with corrected *p*-values < 0.05. (D) GSVA of Notch1 gene sets between WT and KO groups (n = 3 mice/group). In total, there were 166 Notch1 signaling pathway-related genes.

**Additional Figure S2**


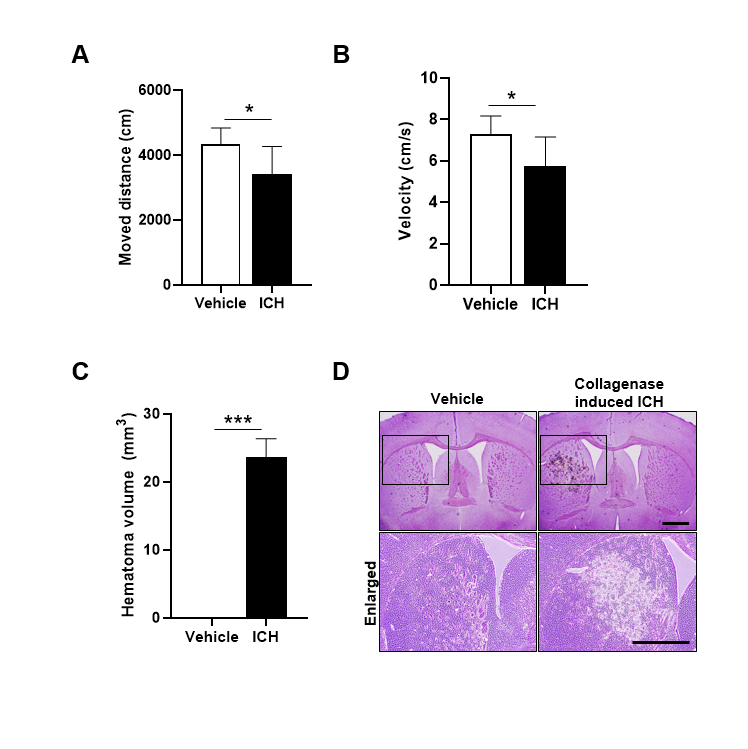


**Additional Figure S2. Characteristics of the ICH mouse model.**

(A, B) Moved distance and movement velocity in an open-field test, used to evaluate general movement (n = 10 mice/group). (C) Hematoma volume, quantified using ImageJ. (D) Representative cresyl violet-stained brain sections showing lesion volume. Scale bar: 100 µm. Data are presented as means ± SD from three independent experiments performed under the same conditions (*P < 0.05, **P < 0.01, ***P < 0.001 vs. Vehicle).

**Additional Figure S3**

**
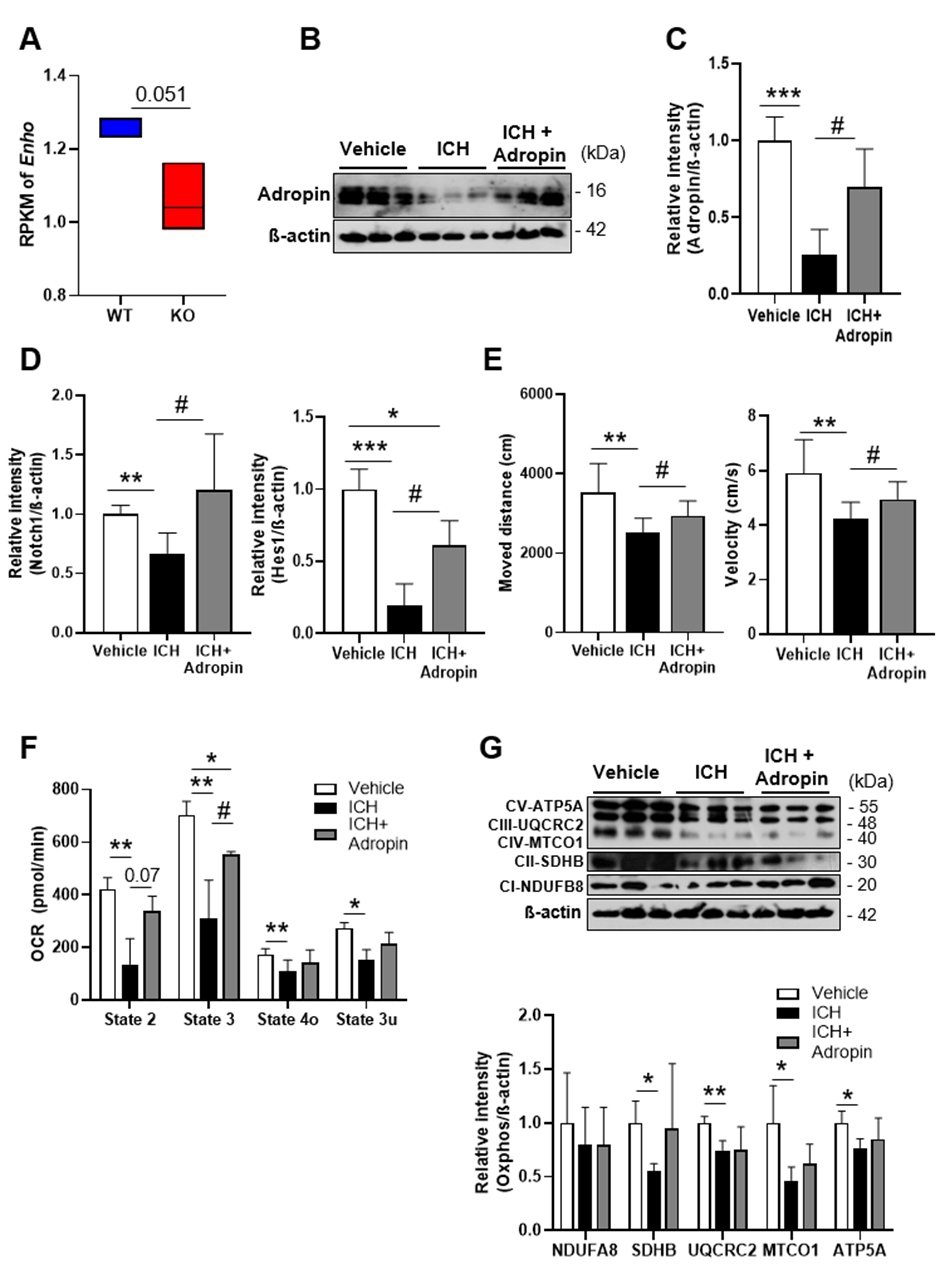
**

**Additional Figure S3. Rescue of ICH pathology by injection of adropin.**

(A) RPKM value for the *Enho* gene from a transcriptomic analysis of TEKCRIF1 KO mice. (B, C) Adropin protein levels in ICH-only and adropin-treated ICH groups. (D) Quantification of Notch1 and Hes1 protein levels in Figure 5B (n=6 mice/group). (E) Moved distance and movement velocity in the open-field test, used to evaluate general movement (n = 11 mice/group). (F) OCR analysis values. State 2: basal respiration; State 3: after ADP injection; State 4o: after oligomycin injection; State 3u: after CCCP injection in the striatum following adropin injection in ICH model mice. (H) Total OxPhos complex protein levels (n = 4 mice/group). Data are presented as means ± and SD from three independent experiments performed under the same conditions (*P < 0.05, **P < 0.01, ***P < 0.001, Vehicle vs ICH; ^#^P < 0.05, ICH vs. ICH+adropin).

**\**

**Additional Figure S2**

**
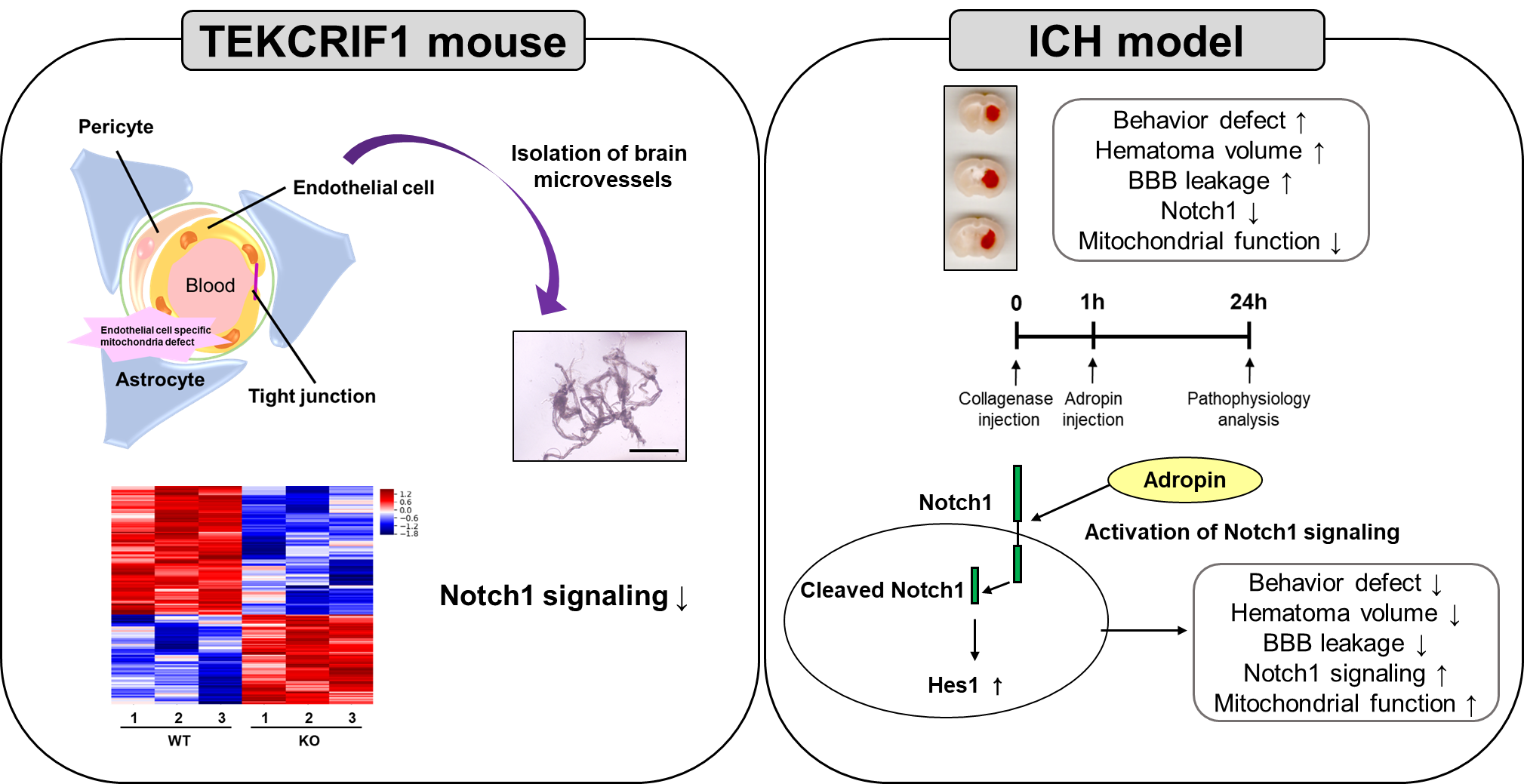
**

**Additional Figure S4. Schematic summary**

Notch1 signaling pathway as a therapeutic target for BBB maintenance by transcriptomic analysis of cerebral microvessels in TEKCRIF1 KO mice, which exhibit a mitochondrial defect in ECs. Activation of Notch1 signaling through treatment with adropin reduced ICH pathologies, including BBB leakage, in association with attenuation of mitochondrial function in brain tissue.
