# Supplementary material for: A transcriptomic analysis of cerebral microvessels reveals the involvement of Notch1 signaling in endothelial mitochondrial-dysfunction-dependent BBB disruption
Source: Fluids Barriers CNS. 2022 Aug 26;19:64. doi: 10.1186/s12987-022-00363-7 (PMC9414148; doi:10.1186/s12987-022-00363-7)
Supplement: Supplementary file 2 — Additional file 2: Table S1. Information of Antibodies. Table S2. List of upstream regulators related to Crif1 deletion in ECs, determined by IPA. [file 12987_2022_363_MOESM2_ESM.docx]

**Additional Table S1. Information of Antibodies**

| Antibodies | Source | Concentration |
| --- | --- | --- |
| Mouse anti-Crif1 | Santa Cruz Biotechnology,  sc-134882 | 1:1000 |
| Rabbit anti-Notch1 | Cell signaling,  3608 | 1:1000 |
| Mouse anti-Hes1 | Santa Cruz Biotechnology,  sc-166410 | 1:500 |
| Total OXPHOS complex | Abcam,  ab110413 | 1:1000 |
| Rabbit anti-Adropin | Novus Biologicals,  NBP1-26387 | 1:500 |
| Mouse anti-β-actin | Santa Cruz Biotechnology,  sc-47778 | 1:1000 |
| Goat anti-Mouse IgG-HRP | Sigma-Aldrich,  12-349 | 1:1000 |
| Goat anti-Rabbit IgG-HRP | Sigma-Aldrich,  12-348 | 1:1000 |

**Additional Table S2. List of upstream regulators related to *Crif1* deletion in ECs, determined by IPA**

| **Upstream Regulator** | **Expr Log Ratio** | **Molecule Type** | **Predicted Activation State** | **Activation z-score** | **Bias Term** | **Bias-corrected z-score** | **p-value of overlap** |
| --- | --- | --- | --- | --- | --- | --- | --- |
| TP53 | -0.333 | transcription regulator | Inhibited | -2.011 | -0.004 | -1.979 | 0.000158 |
| NOTCH1 | -0.285 | transcription regulator | Inhibited | -2.379 | -0.004 | -2.364 | 0.0337 |
| EGFR | -0.265 | kinase | Inhibited | -2.005 | -0.017 | -1.925 | 0.0000569 |
| GABA |  | chemical - endogenous mammalian | Inhibited | -2.401 | 0.031 | -2.581 | 0.00000304 |
| MIR17HG |  | other | Activated | 2.068 | 0.027 | 1.965 | 0.0000146 |
| AGT |  | growth factor | Inhibited | -2.631 | -0.017 | -2.514 | 0.0000372 |
| 8-bromo-cAMP |  | chemical reagent | Inhibited | -2.182 | -0.028 | -2.021 | 0.0000447 |
| camptothecin |  | chemical drug | Inhibited | -2.256 | -0.011 | -2.208 | 0.00119 |
| WNT1 |  | cytokine | Inhibited | -2.621 | -0.024 | -2.559 | 0.00151 |
| SPDEF |  | transcription regulator | Activated | 2.449 | 0.023 | 2.393 | 0.00181 |
| CDKN2A |  | transcription regulator | Inhibited | -2.247 | 0.003 | -2.26 | 0.00212 |
| ATP2A2 |  | transporter | Activated | 2 | 0.018 | 1.963 | 0.00435 |
| AXIN1 |  | other | Activated | 2 | 0.017 | 1.967 | 0.00435 |
| (+)-MK-801 |  | chemical drug | Inhibited | -2.828 | -0.006 | -2.812 | 0.00449 |
| CHIR 99021 |  | chemical drug | Inhibited | -2.177 | -0.023 | -2.128 | 0.00467 |
| SUCNR1 |  | g-protein coupled receptor | Inhibited | -2 | -0.029 | -1.943 | 0.00518 |
| DGAT1 |  | enzyme | Activated | 2 | -0.012 | 2.023 | 0.00611 |
| epicatechin |  | chemical drug | Inhibited | -2.121 | -0.016 | -2.076 | 0.00706 |
| tretinoin |  | chemical - endogenous mammalian | Inhibited | -2.052 | -0.012 | -1.965 | 0.0109 |
| CD44 |  | other | Inhibited | -2.23 | -0.026 | -2.138 | 0.0115 |
| KRAS |  | enzyme | Activated | 2.309 | -0.001 | 2.314 | 0.0154 |
| KITLG |  | growth factor | Inhibited | -2.345 | -0.028 | -2.26 | 0.0155 |
| IKBKG |  | kinase | Inhibited | -2.041 | -0.024 | -1.966 | 0.0176 |
| Gm15807/Hmgn5 |  | other | Activated | 2 | -0.025 | 2.049 | 0.0219 |
| roscovitine |  | chemical drug | Inhibited | -2.203 | 0.022 | -2.251 | 0.0232 |
| bortezomib |  | chemical drug | Inhibited | -2.46 | -0.004 | -2.446 | 0.0284 |
| MKNK1 |  | kinase | Inhibited | -2.111 | -0.034 | -1.996 | 0.0337 |
| diethylstilbestrol |  | chemical drug | Inhibited | -2.092 | -0.015 | -2.037 | 0.034 |
| PPARGC1A |  | transcription regulator | Activated | 2.784 | -0.008 | 2.82 | 0.0345 |
| molybdenum disulfide |  | chemical reagent | Activated | 2.333 | 0.015 | 2.288 | 0.0406 |
| sodium bisulfide |  | chemical reagent | Activated | 2.221 | 0.004 | 2.212 | 0.0433 |
| JAG1 |  | growth factor | Inhibited | -2.211 | -0.028 | -2.149 | 0.0433 |
| etoposide |  | chemical drug | Inhibited | -2.303 | -0.017 | -2.247 | 0.0452 |
| PTH |  | other | Inhibited | -2.195 | -0.022 | -2.136 | 0.0498 |
